# Lycopene Ameliorates Hypoxic Pulmonary Hypertension via Suppression of Oxidative Stress

**DOI:** 10.1155/2022/9179427

**Published:** 2022-10-07

**Authors:** Dingyou Wang, Yuke Ji, Rui Wang, Ke Cheng, Liang Liu, Na Wu, Qing Tang, Xu Zheng, Junxia Li, Zhilong Zhu, Qinghua Wang, Xueyan Zhang, Runbo Li, Jinjin Pan, Zheng Sui, Yuhui Yuan

**Affiliations:** ^1^The Second Affiliated Hospital, Institute of Cancer Stem Cell, Dalian Medical University, Dalian 116000, China; ^2^Department of Vasculocardiology, The Second Affiliated Hospital of Dalian Medical University, Dalian 116023, China

## Abstract

Hypoxic pulmonary hypertension (HPH) is a progressive cardiopulmonary system disease characterized by pulmonary vascular remodeling. Its occurrence and progression are closely related to oxidative stress. Lycopene, extracted from red vegetables and fruits, exhibits a particularly high antioxidant capacity that is beneficial for cardiovascular diseases. Nevertheless, the role and mechanism of lycopene in HPH remain unknown. Here, we found that lycopene reversed the elevated right ventricular systolic pressure (RVSP), right ventricular hypertrophy, and pulmonary vascular remodeling induced by hypoxia in rats. In vitro, lycopene caused lower proliferation and migration of PASMCs, with higher apoptosis. Consistent with the antiproliferative result of lycopene on hypoxic PASMCs, the hippo signaling pathway associated with cell growth was activated. Furthermore, lycopene reduced malondialdehyde (MDA) levels and enhanced superoxide dismutase (SOD) activity in the lungs and serum of rats under hypoxia conditions. The expression of NOX4 in the lungs was also significantly decreased. Hypoxic PASMCs subjected to lycopene showed decreased reactive oxygen species (ROS) production and NOX4 expression. Importantly, lycopene repressed HIF-1*α* expression both in the lungs and PASMCs in response to hypoxia in the absence of a significant change of HIF-1*α* mRNA. Compared with 2ME2 (a HIF-1*α* inhibitor) alone treatment, lycopene treatment did not significantly change PASMC proliferation, NOX4 expression, and ROS production after 2ME2 blocked HIF-1*α*, suggesting the inhibitory effect of lycopene on HIF-1*α*-NOX4-ROS axis and the targeted effect on HIF-1*α*. After CHX blocked protein synthesis, lycopene promoted the protein degradation of HIF-1*α*. MG-132, a proteasome inhibitor, notably reversed the decrease in HIF-1*α* protein level induced by lycopene in response to hypoxia. Therefore, lycopene suppressed hypoxia-induced oxidative stress through HIF-1*α*-NOX4-ROS axis, thereby alleviating HPH. Our findings will provide a new research direction for clinical HPH therapies.

## 1. Introduction

Pulmonary hypertension (PH) is a cardiopulmonary respiratory system disease characterized by a resting mean pulmonary artery pressure (mPAP) ≥ 25 mmHg measured with a right heart catheterization, accompanied by a pathological hallmark of pulmonary vascular remodeling [1]. The World Health Organization (WHO) classified9r into 5 groups, and hypoxic pulmonary hypertension (HPH) is related to group 3 [2]. HPH-induced pulmonary and cardiac dysfunction is a serious syndrome with high morbidity and mortality [3]. Persistent chronic hypoxia derived from cardiopulmonary system illnesses, including chronic obstructive pulmonary disease, obstructive sleep apnea, and chronic mountain sickness, drive to worsening pulmonary vascular remodeling and increased pulmonary vascular resistance. Eventually, right ventricular failure and premature death occur [4–8]. Recently, there has been a lack of efficient therapeutic methods for HPH, which is limited to vasodilating drugs and surgery. Therefore, it is urgent to find a new treatment for this disease.

Hypoxia exposure causes phenotypical, biochemical, and functional changes in pulmonary arterial cells, such as endothelial cells, smooth muscle cells, and fibroblasts [9]. Among them, excessive proliferation of pulmonary artery smooth muscle cells (PASMCs) leading to medial wall thickening is considered the critical cellular basis for pulmonary vascular remodeling [9]. Numerous pieces of evidence have demonstrated that oxidative stress contributes to PASMC proliferation in hypoxia-induced PH when excessive reactive oxygen species (ROS) are generated [10, 11]. Nicotinamide adenine dinucleotide phosphate (NADPH) oxidase 4 (NOX4) is one of the predominant sources of incremental ROS accumulation that can result in hyperproliferation and migration of PASMCs in HPH [12–14]. HIF-1*α* was reported to be essential in facilitating the increase of NOX4 in PASMCs under hypoxia conditions [14–16]. Thus, targeting HIF-1*α* and NOX4 to reduce oxidative stress potentially serves as a therapeutic method for HPH.

Lycopene is a member of the tetraterpene carotenoid family and is extracted from red vegetables and fruits, such as tomato, pink grapefruit, and gac melons [17]. It has antiproliferative, proapoptotic, antioxidative, and anti-inflammatory activities [17–19]. Intriguingly, a previous study reported that lycopene could be docked to HIF-1*α* modeled protein and inhibit its activity [20]. Lycopene has been proven to play beneficial roles in cardiovascular diseases. These diseases include atherosclerosis, hypertension, myocardial infarction, and stroke [21–26]. However, the effect of lycopene on HPH remains unknown. In the present study, our data demonstrated that lycopene effectively repressed hyperproliferation of PASMCs, thereby preventing vascular remodeling in HPH. The mechanism may inhibit oxidative stress induced by hypoxia via HIF-1*α*-NOX4-ROS axis.

## 2. Materials and Methods

### 2.1. Reagents and Antibodies

Lycopene (PHR1170) used in the animal experiment was purchased from Sigma-Aldrich (Shanghai, China) and was dissolved in 0.5% CMC-Na. Lycopene diluted in 0.5 M NaOH for cell experiment was purchased from Yuanye (Shanghai, China). 2-Methoxyestradiol (2ME2, M125960), MG-132 (M126521), and cycloheximide (CHX, C112766) were purchased from Aladdin (Shanghai, China).

Antibody against HIF-1*α* (ab228649) was purchased from Abcam (Cambridge, USA). Antibodies against HIF-1*α* (20960-1-AP), NOX4 (14347-1-AP), Ki67 (27309-1-AP), *α*-SMA (55135-1-AP), vWF (27186-1-AP), and PCNA (24036-1-AP) were obtained from Proteintech (Wuhan, China). Antibodies against YAP/TAZ (8418), phospho-YAP (Ser127) (13008), MST1 (3682), LATS1 (3477), MOB1 (13730), CyclinD1 (2978), CyclinD3 (2936), cleaved-caspase 7 (8438), cleaved-caspase 9 (7237), *β*-catenin (8480), and HRP-conjugated goat anti-rabbit/mouse IgG (H+L) (7074/7076) were purchased from Cell Signaling Technology (MA, USA). Antibodies against PCNA (WL03213), Ki67 (WL01384a), and HIF-1*α* (WL01607) were purchased from Wanleibio (Shenyang, China).

### 2.2. Animals and Experimental Design

Thirty-two male Sprague-Dawley rats (100-120 g) were obtained from the Animal Centre of Dalian Medical University (Dalian, China), and the HPH rat model was established as previously reported [27–29]. All experiments were conducted at the SPF (specified pathogen free) Animal Experiment Center of Dalian Medical University and approved by the Institutional Animal Care and Use Committee of the Dalian Medical University. All animals were randomly divided into 4 groups, 8 rats per group: group A, normoxia puls vehicle; group B, normoxia plus lycopene; group C, hypoxia plus vehicle; and group D, hypoxia plus lycopene. The rats in normoxia groups were kept in normobaric room air. The rats in hypoxia groups were housed in normobaric hypoxia exposure (10% O_2_, 10 h/day) for 4 weeks. Lycopene was orally administrated at a dosage of 10 mg/kg every day from 1 week prior to hypoxia for 5 weeks.

### 2.3. Hemodynamic and Right Ventricular Hypertrophy Measurement

The rats were anesthetized with 7% (*m*/*v*) pentobarbital sodium (30 mg/kg). A customized polyethylene catheter, prefilled with 0.6% sodium heparin, was connected to a pressure transducer through the right jugular vein into the right ventricle. The RVSP was recorded by BL-420S Biological Function Experiment System (Chengdu Techman Software, Chengdu, China). Next, the thorax was exposed, and 150 mL of saline was injected through the right ventricle to flush out the blood from the lungs and hearts. Then, the left lung lobes were removed and frozen, and the right lung lobes were fixed with a 4% paraformaldehyde solution. The hearts of half of the rats per group were dissected into the right ventricle (RV), left ventricle, and septum (LV+S). The hearts of other rats were fixed entirely in 4% paraformaldehyde solution. The right ventricular hypertrophy was evaluated by the Fulton index (RV/(LV+S)) and the weight ratio of the right ventricle to body weight (BW) (RV/BW) [29].

### 2.4. Histomorphometric Analysis

After 4% paraformaldehyde fixation for 72 h, the lungs and hearts were embedded with paraffin and sectioned into 4 *μ*m thickness to perform hematoxylin and eosin (H&E) staining as well as elastic Van Gieson (EVG) staining as our previous report [29]. Five pulmonary vessels (25-100 *μ*m external diameter) from each pulmonary section were randomly selected using the microscope (Carl Zeiss, Oberkochen, Germany) and analyzed by Image-Pro Plus, version 7.0 (Media Cybernetics, Maryland, USA). The percent of medial wall area ((medial wall area)/(total vessel area]) × 100, WA%) and the wall thickness (WT) were calculated to assess pulmonary vascular remodeling. The panoramic scan of H&E-stained transverse heart histology sections was used to estimate right ventricular hypertrophy by CaseViewer, version 2.4 (3DHISTECH, Budapest, Hungary).

### 2.5. Immunohistochemistry and Immunofluorescence Detection

For immunohistochemistry (IHC) detection, the lung sections were processed as previously reported [27] and incubated with primary antibodies overnight at 4°C, recognizing, respectively: *α*-SMA (1 : 1200 dilution), vWF (1 : 200 dilution), and PCNA (1 : 500 dilution). Next, biotinylated anti-mouse/rabbit IgG antibodies (1 : 100 dilution) were used before DAB staining. Finally, the lung sections were counterstained lightly with hematoxylin.

For immunofluorescence (IF) detection, the lung sections were incubated with NOX4 (1 : 200 dilution) and HIF-1*α* (1 : 500 dilution) overnight at 4°C. Then, anti-mouse/rabbit IgG conjugated with Alexa Fluor 488/594 dye was cotreated for 2 h at room temperature in the dark. DAPI (4,6-diamidino-2-phenylindole) was used to label the cell nuclei. Five pulmonary vessels (25-100 *μ*m external diameter) from each section were randomly selected using the microscope or fluorescence microscope (Carl Zeiss). The images were analyzed by Image-Pro Plus, Version 7.0.

### 2.6. Malondialdehyde (MDA) and Superoxide Dismutase (SOD) Measurement

The levels of MDA (S0131S, Beyotime, Beijing, China) and SOD (KGT00100-1, KeyGEN, Suzhou, China) in lung tissues and serum of the rats were measured by assay kits. The detailed operation was carried out in strict accordance with the manufacturer's instructions, and the integrated optical density value was detected by an absorbance reader (Perkin Elmer, MA, USA) [27].

### 2.7. Primary Cell Culture and Treatment

The primary PASMCs were isolated, identified, and maintained as previously reported [27–29]. In the study, PASMCs were used between 3 and 6 passages and exposed to normoxia or hypoxia (1% O_2_) for 48 h. Lycopene (1 *μ*M) and MG-132 (500 nM) were administrated. 2ME2 (10 *μ*M, 2 h) was treated prior to hypoxia.

### 2.8. Immunofluorescence Assay in PASMCs

PASMCs (2 × 10^4^ per well) were incubated with anti-Ki67 (1 : 1000 dilution) in 12-well plates overnight at 4°C, followed by cotreatment with anti-rabbit IgG conjugated with Alexa Fluor 488 dye for 2 h. DAPI was used to label the cell nuclei. Five views (at least 300 nuclei were counted) from each group were selected using the fluorescence microscope. The percentage of positive cells to the total number of cells was calculated [29].

### 2.9. CCK8 Assay

Cell viability was detected by a cell counting 8 (CCK8) assay kit (BMU296, Abbkine, Wuhan, China). After normoxia or hypoxia exposure for 48 h, PASMCs were added with 10 *μ*L CCK8 solution and incubated at 37°C for 4 h. The optical density value was detected at 450 nm by an absorbance reader.

### 2.10. Intracellular ROS Measurement

The intracellular ROS was measured by 2,7′-dichlorodihydrofluoresceindiacetate (DCF-DA) (KGAF018, KeyGEN). PASMCs were inoculated with DCF-DA (50 *μ*M) at 37°C for 40 min sheltered from light. Five views (at least 300 nuclei were counted) from each group were selected under the fluorescence microscope. The immunofluorescence intensity was quantitated.

### 2.11. EdU and Hoechst 33342 Staining

The kFluor 488 Click-iT EdU (5-ethynyl-2′-deoxyuridine) and Hoechst 33342 Kit (KGA331, KeyGEN) was used to detect the proliferation and apoptosis of PASMCs. PASMCs were incubated with EdU (50 *μ*M) and Hoechst 33342 (5 *μ*M) at 37°C for 30 min sheltered from light. Five views (at least 300 nuclei were counted) from each group were chosen under the fluorescence microscope. EdU was excited at 495 nm and emitted at 520 nm. Hoechst 33342 was excited at 350 nm and emitted at 461 nm. The percentage of positive cells to the total number of cells was calculated [29].

### 2.12. Cell Cycle Analysis

After dissociated using 0.25% trypsin and washed twice with PBS, PASMCs were fixed with ice-cold 75% ethanol at -20°C for 4 h. The cell cycle analysis was operated with the cell cycle assay kit (WLA010, Wanleibio), and the detailed operation was carried out in strict accordance with the manufacturer's instructions. The cell cycle data was obtained using the FACSCalibur Flow Cytometer (BD Biosciences, NJ, USA). Further analysis was processed by FlowJo (BD Biosciences) [29].

### 2.13. Wound Healing Assay

The migration of PASMCs was detected by wound healing assay. PASMCs (5 × 10^4^) were cultured in 6-well plates for 24 h to form a cell monolayer. After mitomycin treatment for 2 h, the cell monolayer was scratched straightly with a sterile pipette. The acceleration of migration was recorded with the microscope (Leica, Solms, Germany) at 0 h, 12 h, and 24 h. The images were analyzed by Image-Pro Plus, version 7.0 [29].

### 2.14. Western Blot Analysis

The frozen lung tissues and PASMCs were lysed sufficiently in ice-cold Radio Immunoprecipitation Assay (RIPA) buffer with protease and phosphatase inhibitors. The protein content was determined by a bicinchoninic acid protein assay (BCA) kit (P0012S, Beyotime). Then, 30 *μ*g of protein for the lung tissues and 20 *μ*g of protein for the PASMCs were used. After being blocked with 5% skim milk, the PVDF membranes were incubated with the primary antibodies against HIF-1*α* (1 : 5000 dilution), NOX4 (1 : 1000 dilution), Ki67 (1 : 500 dilution), cleaved-caspase 7 (1 : 2000 dilution), cleaved-caspase 9 (1 : 2000 dilution), Cyclin D1 (1 : 1000 dilution), Cyclin D3 (1 : 1000 dilution), P27 (1 : 1000 dilution), *β*-catenin (1 : 1000 dilution), MST1 (1 : 1000 dilution), LATS1(1 : 1000 dilution), MOB1 (1 : 1000 dilution), YAP/TAZ (1 : 1000 dilution), phospho-YAP (1 : 1000 dilution), and *β*-actin (1 : 5000 dilution). The signals were detected by the enhanced chemiluminescent (ECL) kit (BMU102-CN, Abbkine). The relative quantitation was calculated by Image Lab (Bio-Rad Laboratories, CA, USA) [27, 28].

### 2.15. RT-Quantitative PCR Detection

Total RNA extraction and RT-quantitative PCR (qPCR) were performed as in our previous report [28]. Primer pairs for HIF-1*α* PCR were (forward) 5′-ACCCTCTGATTTAGCATGTAG-3′, (reverse) 5′-GTAGGTTCTGCTGCCTTGT3′ and for housekeeping gene *β*-actin (forward) 5′-AGTCCCTCACCCTCCCAAAAG-3′, (reverse) 5′-AAGCAATGCTGTCACCTTCCC-3′.

### 2.16. Protein Degradation Experiment

PASMCs (1 × 10^7^) were treated with lycopene or vehicle under hypoxia conditions for 48 h. After adding cyclohexanone (12.5 mg/mL), lysates were harvested at 0 h, 2 h, 4 h, and 6 h. The expression of HIF-1*α* was detected by western blot, and the decay rate was determined [29].

### 2.17. Statistics

All results were presented as mean ± S.E.M and analyzed using SPSS, version 20.0 (IBM, Chicago, USA). Differences between groups were compared by one-way ANOVA after passing normality and equal variance tests. *P* < 0.05 was considered significant difference. The graphs were plotted using GraphPad Prism, version 8.02 (GraphPad Software, CA, USA), and figures were generated with Adobe Illustrator, version CC 2019 (Adobe, California, USA).

## 3. Results

### 3.1. Lycopene Attenuated Hypoxia-Induced Pulmonary Hypertension in Rats

**Figure 1:**
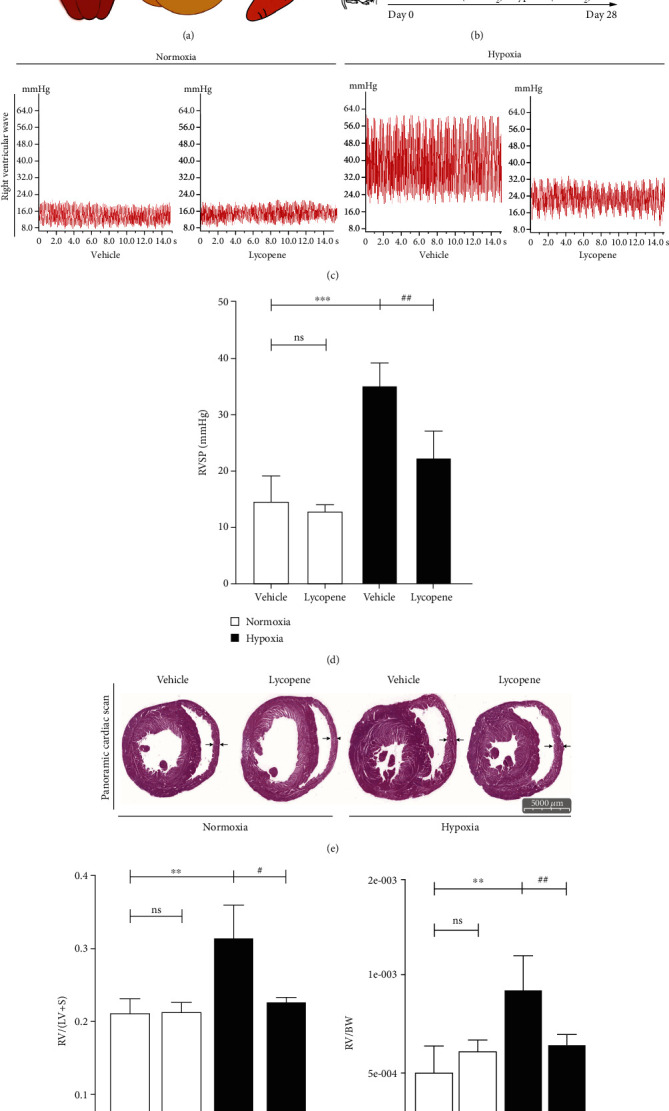
Lycopene attenuated hypoxia-induced pulmonary hypertension in the HPH rat model. (a) The molecular structure of lycopene, foods rich in lycopene, and the criteria of oral bioavailability and drug-like properties of lycopene. (b) The animal treatment design and groups are presented. (c) The representative images of right ventricular systolic pressure (RVSP) waveform in rats. (d) The quantification of RVSP in rats. (e) The panoramic views of the histological sections of transverse hearts in rats. Scale bars: 5000 *μ*m. (f) The weight ratio of the right ventricular (RV) to the left ventricular plus septum (LV+S) in rats. (g) The weight ratio of RV to body weight (BW) in rats. Values are means ± S.E.M (*n* = 4 or 8). ^∗^*P* < 0.05, ^∗∗^*P* < 0.01, and ^∗∗∗^*P* < 0.001 vs. the normoxia group; ^#^*P* < 0.05, ^##^*P* < 0.01 vs. the hypoxia group.

As shown in Figure 1(a), the molecular formula of lycopene is C40H56 with a chemical structure that contains 11 conjugated double bonds and 2 nonconjugated double bonds, constituting a straight-chain hydrocarbon. This particular structure gives lycopene a high antioxidative activity [21]. In addition, the availability of lycopene has been identified to meet the criteria of OB (oral bioavailability) ≥ 30% and DL (drug‐like properties) ≥ 0.18 by the Traditional Chinese Medicine Systems Pharmacology (TCMSP, updated on May 31, 2014) [30].

Aiming to detect the role of lycopene in HPH in vivo, Sprague Dawley (SD) rats were exposed to hypoxia (10% O_2_) for 4 weeks and orally administrated with lycopene 10 mg/kg daily (Figure 1(b)). The hemodynamic results showed that lycopene administration was sufficient to alleviate the increased RVSP induced by hypoxia (Figures 1(c) and 1(d)). Lycopene also significantly decreased the Fulton index (RV/(LV+Septum)) and RV/BW, indicating that right ventricular hypertrophy was prevented by lycopene in the HPH rat model (Figures 1(e) and 1(f)).

### 3.2. Lycopene Suppressed Hypoxia-Induced Pulmonary Vascular Remodeling in Rats

**Figure 2:**
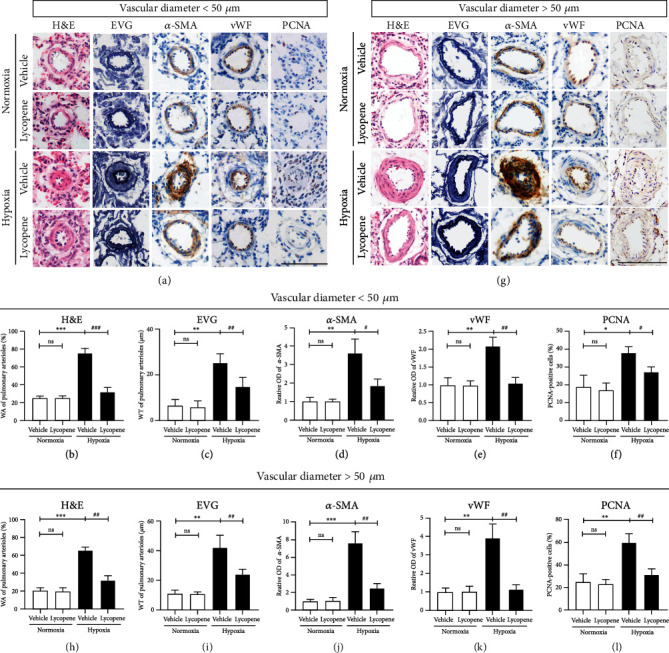
Lycopene suppressed hypoxia-induced pulmonary vascular remodeling in the HPH rat model. (a) Hematoxylin and eosin (H&E) staining, Verhoeff's van Gibson (EVG) staining, and immunohistochemistry staining with *α*-SMA, vWF, and PCNA in the pulmonary arterioles with a diameter less than 50 *μ*m. Scale bars: 50 *μ*m. (b–f) The quantification of the percentage of medial wall area (WA%), arterial wall thickness (WT), relative optical density (OD) value of *α*-SMA and vWF, and percentage of PCNA-positive cells in the pulmonary arterioles with a diameter less than 50 *μ*m. (g) H&E staining, EVG staining, and immunohistochemical staining of *α*-SMA, vWF, and PCNA in the pulmonary arterioles with a diameter larger than 50 *μ*m. Scale bars: 100 *μ*m. (h, i) The quantification of WA%, WT, OD value of *α*-SMA and vWF, and percentage of PCNA-positive cells in the pulmonary arterioles with a diameter larger than 50 *μ*m. Values are means ± S.E.M (*n* = 8). ^∗^*P* < 0.05, ^∗∗^*P* < 0.01, and ^∗∗∗^*P* < 0.001 vs. the normoxia group; ^#^*P* < 0.05, ^##^*P* < 0.01, and ^###^*P* < 0.001 vs. the hypoxia group.

We next detected the effect of lycopene in hypoxia-induced pulmonary vascular remodeling. The WA% and WT were calculated in the pulmonary arterioles with a diameter less than 50 *μ*m and larger than 50 *μ*m. Hypoxia significantly enhanced the WA% and WT in rats, whereas these changes in the lycopene-treated rats were decreased under hypoxia exposure (Figures 2(a)–2(c) and 2(g)–2(i)). Furthermore, the remodeling of the medial wall of pulmonary arterioles was detected using an *α*-SMA antibody. As expected, the *α*-SMA-positive area in the pulmonary arterioles was significantly increased in rats after hypoxia exposure. However, lycopene decreased the positive areas (Figures 2(a), 2(d), 2(g), and 2(j)). Then, the endothelial layer in pulmonary arterioles of rats was detected by immunohistochemical staining with a vWF antibody. We found that hypoxia-induced excess vWF-positive area in the pulmonary arterioles was reduced in the lycopene-treated group (Figures 2(a), 2(e), 2(g), and 2(k)). The high percentage of PCNA-positive cells in pulmonary arterioles was also lowered in lycopene-treated rats compared to the control group after hypoxia exposure (Figures 2(a), 2(f), 2(g), and 2(l)). These data demonstrated that lycopene alleviated hypoxia-induced pulmonary vascular remodeling in HPH.

### 3.3. Lycopene Inhibited Hyperproliferation and Promoted Apoptosis of Hypoxic PASMCs

**Figure 3:**
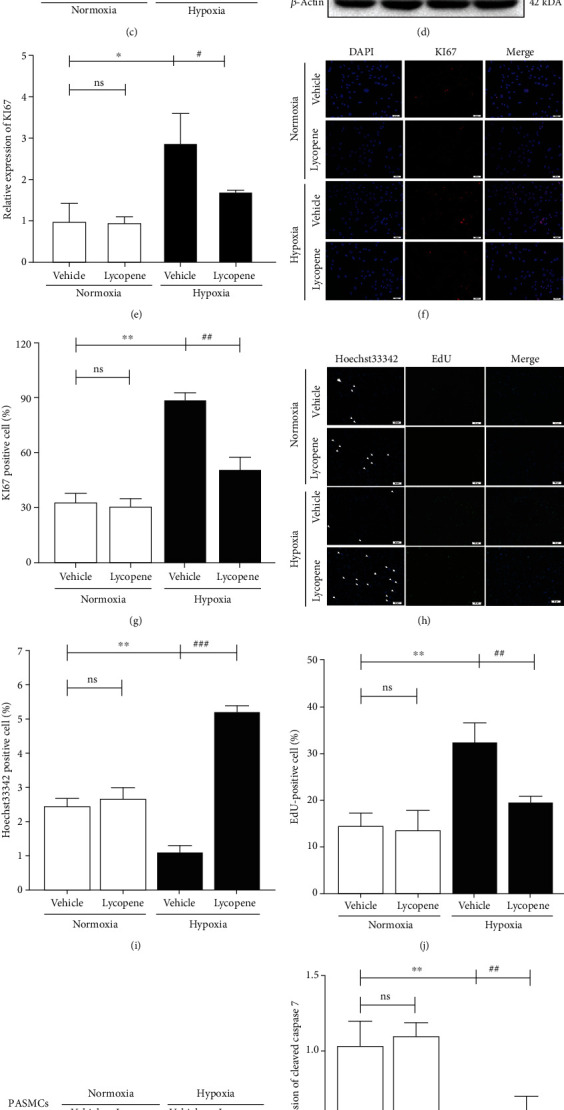
Lycopene inhibited hyperproliferation and promoted apoptosis of PASMCs in response to hypoxia. (a) The viability of PASMCs was determined by cell counting kit 8 (CCK-8) assay. (b, d) Ki67 expression in lungs of rats and PASMCs was detected after lycopene treatment by western blot. *β*-Actin was used as a loading control. (c, e) The quantification of Ki67 protein levels in lungs and PASMCs. (f) IF staining for Ki67 (in red) in PASMCs. Cell nuclei were counterstained with DAPI (in blue). Scale bars: 50 *μ*m. (g) The quantification of the ratio of Ki67-positive PASMCs. (h) EdU staining was used to label cells in DNA synthesis (in green). And Hoechst 33342 staining was used to label cells in the process of apoptosis. (i, j) The quantification of the percentage of EdU-positive cells and Hoechst 33342-positive cells. (k) Cleaved-caspase 7 and cleaved caspase-9 expressions in PASMCs were detected by western blot. *β*-Actin was used as a loading control. (l, m) The quantification of cleaved-caspase 7 and cleaved-caspase 9 protein levels in PASMCs. Values are means ± S.E.M (*n* = 3-5). ^∗^*P* < 0.05, ^∗∗^*P* < 0.01, and ^∗∗∗^*P* < 0.001 vs. the normoxia group; ^#^*P* < 0.05, ^##^*P* < 0.01, and ^###^*P* < 0.001 vs. hypoxia group.

To explore the cellular basis of pulmonary vascular remodeling, the primary PASMC viability was examined by a CCK-8 assay. The results showed that lycopene reduced hypoxia-induced PASMC hyperproliferation in a dose-dependent manner (Figure 3(a)). Then, lycopene (1 *μ*M) was chosen for further investigation. Hypoxia upregulated the expression of Ki67 in PASMCs and lung tissues; however, the upregulation was lowered after lycopene treatment (Figures 3(b)–3(g)). EdU staining results further confirmed the antiproliferative effect of lycopene (Figures 3(h) and 3(i)). In addition, Hoechst 33342 staining data showed a reduction of apoptosis in PASMCs under hypoxia conditions; nevertheless, lycopene reversed the decline (Figures 3(h) and 3(j)). The expression of cleaved-caspase 9 and cleaved-caspase 7 was enhanced in lycopene-treated PASMCs compared with vehicle-treated PASMCs under hypoxia conditions (Figures 3(k)–3(m)). These data suggested that lycopene suppressed hyperproliferation and promoted apoptosis in PASMCs under hypoxia conditions.

### 3.4. Lycopene Arrested Acceleration of the Cell Cycle in PASMCs Exposed to Hypoxia

**Figure 4:**
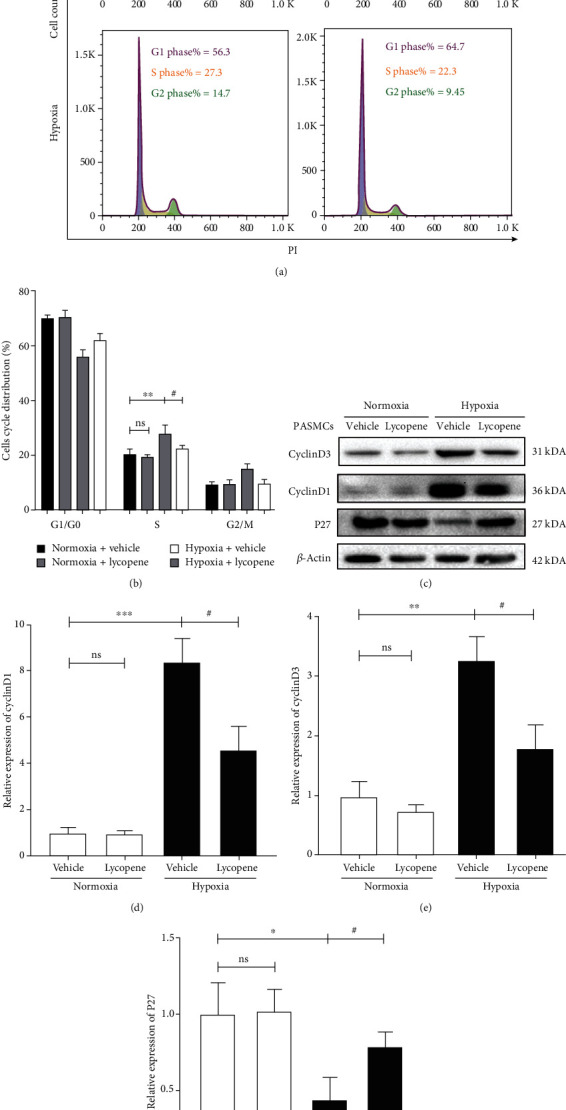
Lycopene arrested the acceleration of cell cycle in PASMCs under hypoxia conditions. (a) The cell cycle of PASMCs was performed by flow cytometry analysis. Pyrimidine iodide (PI). (b) Quantification of the percentage of PASMCs at each cell cycle phase. (c) Cyclin D3, Cyclin D1, and P27 expressions in PASMCs were detected by western blot. *β*-Actin was used as a loading control. (d–f) The quantification of Cyclin D3, Cyclin D1, and P27 protein levels in PASMCs. Value are means ± S.E.M (*n* = 3-5). ^∗^*P* < 0.05, ^∗∗^*P* < 0.01, and ^∗∗∗^*P* < 0.001 vs. the normoxia group; ^#^*P* < 0.05 vs. the hypoxia group.

Then, the cell cycle of PASMCs was assessed by flow cytometry analysis. The data revealed that lycopene treatment reduced the percentage of cells in the S phase under hypoxia conditions, indicating a cell cycle arrest at G1/S phase (Figures 4(a) and 4(b)). We next examined the expression of crucial proteins that participate in cell cycle (Figures 4(c)–4(f)). The results demonstrated that hypoxia remarkably increased the expression of cyclin D1 and cyclin D3, with decreased expression of P27. In contrast, the expression of these proteins was significantly reversed by lycopene in response to hypoxia. These data indicated that lycopene suppressed the cycle acceleration of PASMCs exposed to hypoxia.

### 3.5. Lycopene Inhibited PASMC Migration under Hypoxia Conditions

**Figure 5:**
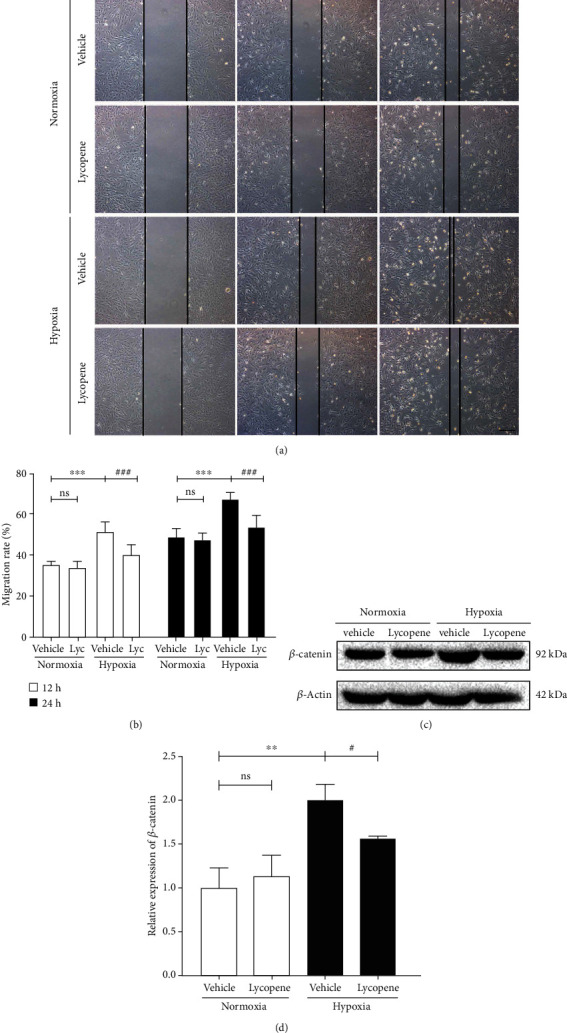
Lycopene inhibited PASMC migration under hypoxia conditions. (a) The migration of PASMCs was detected by wound healing assay. Scale bars: 200 *μ*m. (b) The quantification of the migration rate in PASMCs. (c) *β*-Catenin expression in PASMCs was detected by western blot. *β*-Actin was used as a loading control. (d) The quantification of *β*-catenin protein levels in PASMCs. Value are means ± S.E.M (*n* = 3-5). ^∗∗^*P* < 0.01, ^∗∗∗^*P* < 0.001 vs. the normoxia group; ^#^*P* < 0.05, ^###^*P* < 0.001 vs. the hypoxia group.

We next detected the effect of lycopene in the migration of PASMCs under hypoxia conditions. The wound healing assay showed that lycopene attenuated hypoxia-induced migration rate of PASMCs (Figures 5(a) and 5(b)). The expression of *β*-catenin was upregulated under hypoxia conditions. However, the increased expression of *β*-catenin was reduced by lycopene (Figures 5(c) and 5(d)). These data indicated that lycopene suppressed the migration of PASMCs under hypoxia conditions.

### 3.6. Lycopene Activated Hippo Signaling Pathway in Response to Hypoxia In Vivo and In Vitro

**Figure 6:**
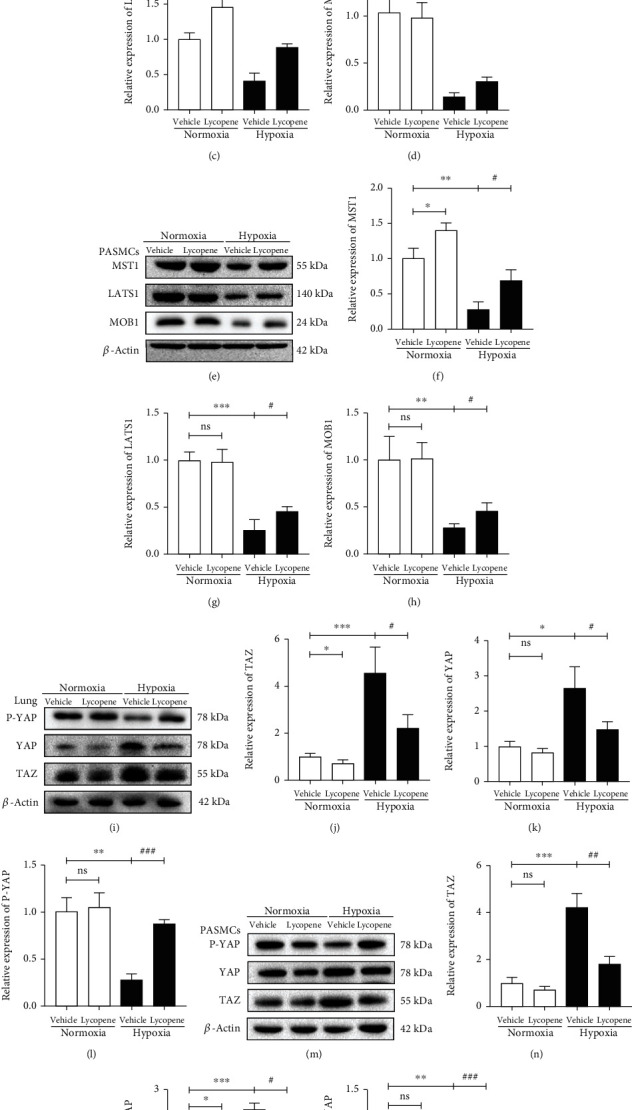
Lycopene activated hippo signaling pathway in response to hypoxia in vivo and in vitro. (a) MST1, LATS1, and MOB1 protein expressions in lungs of rats were detected by western blot. *β*-Actin was used as a loading control. (b–d) The quantification of MST1, LATS1, and MOB1 protein levels in the lungs. (e) MST1, LATS1, and MOB1 protein expression in PASMCs were detected by western blot. *β*-Actin was used as a loading control. (f–h) The quantification of MST1, LATS1, and MOB1 protein levels in PASMCs. (i) YAP, TAZ, and P-YAP protein expressions in the lungs of rats were detected by western blot. *β*-Actin was used as a loading control. (j–l) The quantification of YAP, TAZ, and P-YAP protein levels in lungs. (m) YAP, TAZ, and P-YAP protein expressions in PASMCs were detected by western blot. *β*-Actin was used as a loading control. (n–p) The quantification of YAP, TAZ, and P-YAP protein levels in PASMCs. Values are means ± S.E.M (*n* = 3). ^∗^*P* < 0.05, ^∗∗^*P* < 0.01, and ^∗∗∗^*P* < 0.001 vs. the normoxia group; ^#^*P* < 0.05, ^##^*P* < 0.01, and ^###^*P* < 0.001 vs. the hypoxia group.

Several studies have reported that the hippo signaling pathway was involved PASMC proliferation, apoptosis, and migration in HPH [31–36]. We investigated whether the inhibitory effect of lycopene on vascular remodeling was regulated by hippo pathway. The western blot results showed that the expression of MST1 was decreased in lung tissues and PASMCs in response to hypoxia; nevertheless, lycopene reversed MST1 reduction (Figures 6(a), 6(b), 6(e), and 6(f)). Next, the downstream proteins LAST1, MOB1, YAP, and TAZ were further detected. The levels of LATS1 and MOB1 lowered significantly under hypoxia conditions. However, the changes were reversed following lycopene treatment (Figures 6(a), 6(e), 6(c), 6(d), and 6(g)–6(f)). Furthermore, chronic hypoxia notably increased the YAP and TAZ protein levels and decreased phosphorylation of YAP. After lycopene treatment, the expression of these proteins was significantly reversed (Figures 6(i)–6(p)). These findings suggested that lycopene activated the hippo signaling pathway in response to hypoxia.

### 3.7. Lycopene Alleviated Hypoxia-Induced Oxidative Stress via the HIF-1*α*-NOX4-ROS Axis

**Figure 7:**
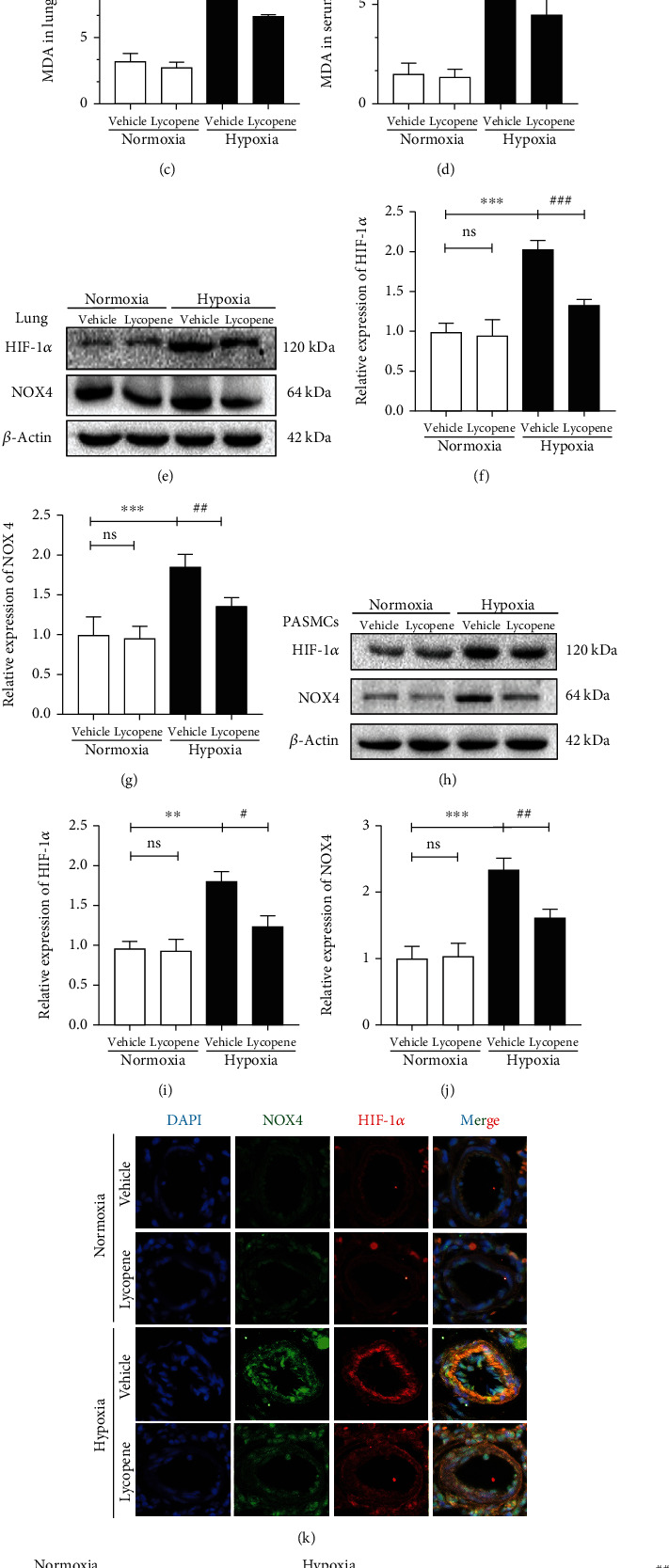
Lycopene alleviated hypoxia-induced oxidative stress. (a, b) The levels of SOD in lungs and serum of rats. (c, d) The levels of MDA in lungs and serum of rats. (e) HIF-1*α* and NOX4 expressions in the lungs of rats were detected by western blot. *β*-Actin was used as a loading control. (f, g) The quantification of HIF-1*α* and NOX4 protein levels in lungs of rats. (h) Immunofluorescence staining for HIF-1*α* (in red) and NOX4 (in green) in lung tissues of rats. Cell nuclei are counterstained with DAPI (in blue). Scale bars: 50 *μ*m. (i) HIF-1*α* and NOX4 expressions in PASMCs were detected by western blot. *β*-Actin was used as a loading control. (j, k) The quantification of HIF-1*α* and NOX4 protein levels in PASMCs. (l, m) The intracellular ROS was detected in PASMCs by the DCFH-DA assay kit. Values are means ± S.E.M (*n* = 3-5). ^∗^*P* < 0.05, ^∗∗^*P* < 0.01, and ^∗∗∗^*P* < 0.001 vs. the normoxia group; ^#^*P* < 0.05, ^##^*P* < 0.01, and ^###^*P* < 0.001 vs. the hypoxia group.

To investigate the mechanism of lycopene in HPH, we detected the oxidative stress induced by hypoxia in vivo and in vitro. Lycopene reduced MDA levels and enhanced SOD activity in serum and lungs of rats exposed to hypoxia (Figures 7(a)–7(d)). Western blot results showed that the increased protein levels of HIF-1*α* and NOX4 under hypoxia conditions were lowered by lycopene, which was further confirmed by immunofluorescence detection (Figures 7(e)–7(h)). Consistent with the in vivo results, lycopene also decreased HIF-1*α* and NOX4 expressions and ROS production in PASMCs (Figures 7(i)–7(m)).

**Figure 8:**
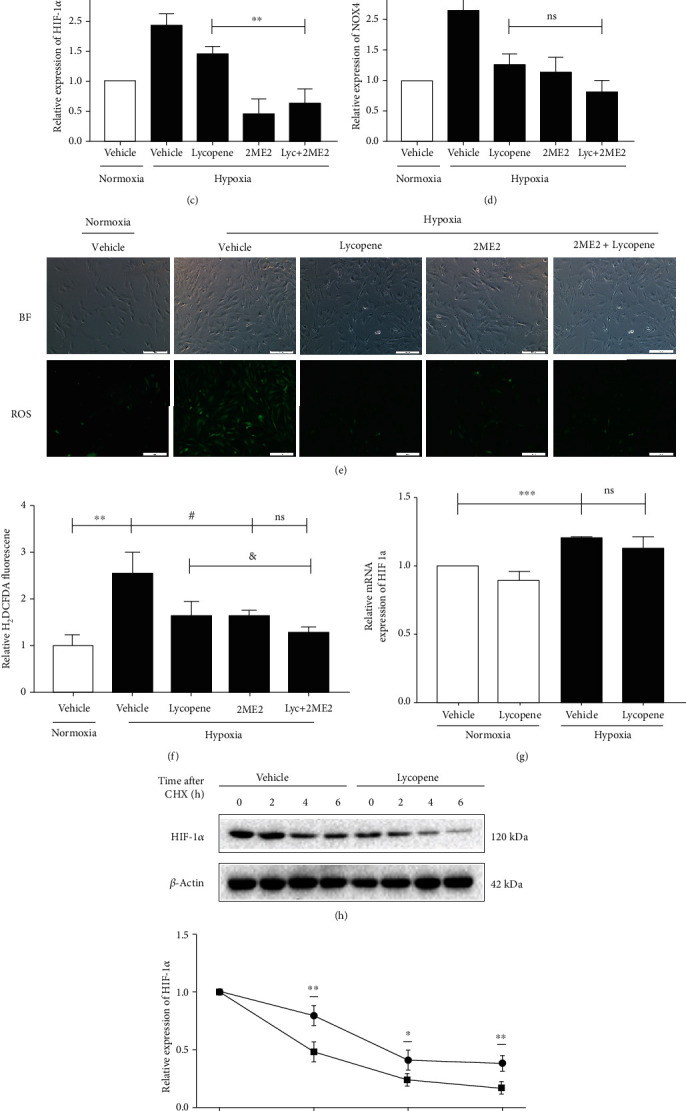
Lycopene regulated HIF-1*α*-NOX4-ROS axis to inhibit oxidative stress in HPH. (a) The cell viability in PASMCs was detected by CCK8 assay after pretreatment with 2ME2 for 2 h and followed by lycopene for 48 h in response to hypoxia. ^∗∗^*P* < 0.01 vs. the normoxia group; ^##^*P* < 0.01, ^###^*P* < 0.001 vs. hypoxia group. (b) The expression of HIF-1*α* and NOX4 in PASMCs pre-treated with 2ME2 for 2 h and followed by lycopene for 48 h was detected by western blot. *β*-Actin was used as a loading control. (c, d) The quantification of HIF-1*α* and NOX4 protein levels in PASMCs. ^∗∗^*P* < 0.01, ^∗∗∗^*P* < 0.001 vs. the normoxia group; ^##^*P* < 0.01 vs. the hypoxia group; ^&&^*P* < 0.01 vs. the hypoxia plus lycopene group. (e, f) The intracellular ROS was detected in PASMCs pretreated with 2ME2 for 2 h and followed by lycopene for 48 h by the DCFH-DA assay kit. ^∗∗^*P* < 0.01 vs. the normoxia group; ^#^*P* < 0.05 vs. the hypoxia group; ^&^*P* < 0.05 vs. the hypoxia plus lycopene group. (g) HIF-1*α* mRNA levels were detected by qPCR. ^∗∗∗^*P* < 0.001 vs. the normoxia group. (h) The expression of HIF-1*α* in PASMCs was detected by western blot at the indicated time points after adding cyclohexanone (12.5 mg/mL). (i) The quantification of the decay rate of HIF-1*α*. ^∗^*P* < 0.05, ^∗∗^*P* < 0.01 vs. the hypoxia group. (l) The expression of HIF-1*α* in PASMCs were detected by western blot after treatment with MG132 (500 nM) or lycopene for 48 h. (m) The quantification of HIF-1*α* protein levels. ^∗∗∗^*P* < 0.001 vs. the normoxia group; ^##^*P* < 0.01 vs. the hypoxia group; ^&&^*P* < 0.01 vs. the hypoxia plus lycopene group. Values are means ± S.E.M (*n* = 3-5). ns: no significance.

We hypothesized that lycopene regulated HIF-1*α*-NOX4-ROS axis to inhibit hypoxia-induced oxidative stress. To confirm this opinion, we pretreated PASMCs with 2ME2, a HIF-1*α* inhibitor, for 2 h for further investigation. Compared to 2ME2 alone treatment, 2ME2 pretreatment plus lycopene treatment could not significantly changed the cell viability of PASMCs exposed to hypoxia (Figure 8(a)). At the same time, there was also no significant difference in HIF-1*α* and NOX4 expressions and ROS production between 2ME2 plus lycopene treatment and 2ME2 alone treatment in response to hypoxia, suggesting the inhibitory effect of lycopene on HIF-1*α*-NOX4-ROS axis and the targeted effect on HIF-1*α* (Figures 8(b)–8(f)). Next, we explored how lycopene targeted HIF-1*α*. The expression of HIF-1*α* gene was detected by qPCR. As shown in Figure 8(g), lycopene did not change HIF-1*α* mRNA levels in response to hypoxia. However, when protein synthesis of PASMCs was blocked by CHX, a broad-spectrum and nonspecific protein synthesis inhibitor, cells treated with lycopene showed lowered protein levels of HIF-1*α* compared with those treated with vehicle in response to hypoxia, which indicated that lycopene promoted HIF-1*α* protein degradation (Figures 8(h) and 8(i)). In addition, MG132, a proteasome inhibitor, reversed the decrease of HIF-1*α* protein levels induced by lycopene under hypoxia conditions (Figures 8(j) and 8(k)). These findings suggested that lycopene inhibited hypoxia-induced oxidative stress via the HIF-1*α*-NOX4-ROS axis and downregulated HIF-1*α* protein levels through the proteasome-dependent degradation pathway in HPH.

## 4. Discussion

**Figure 9:**
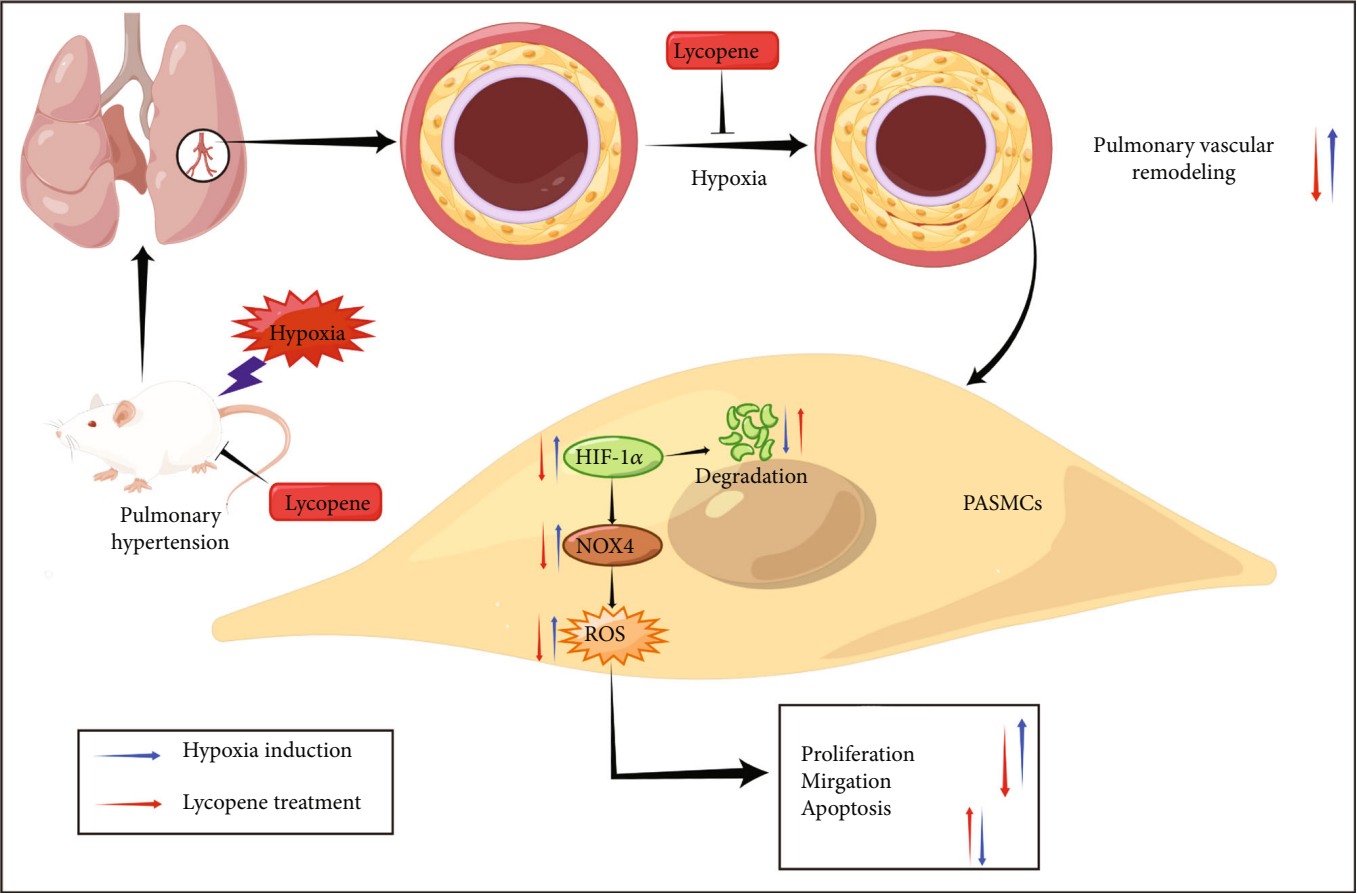
The mechanism of lycopene attenuated hypoxia-induced pulmonary hypertension. Hypoxia causes pulmonary hypertension and pulmonary vascular remodeling in rats that manifests excessive proliferation of PASMCs as its cellular basis. Lycopene could alleviate hypoxia-induced pulmonary vascular remodeling and hyperproliferation of PASMCs. The underlying mechanism may be that lycopene repressed hypoxia-induced oxidative stress via blockading HIF-1*α*-NOX4-ROS axis.

In this study, we successfully established an HPH rat model with increased RVSP, RV hypertrophy, and pulmonary vascular remodeling. Lycopene treatment reversed these pathological changes and attenuated HPH. Moreover, lycopene promoted apoptosis, arrested the acceleration of the cell cycle, and repressed migration in PASMCs, thereby alleviating pulmonary vascular remodeling in HPH. The PASMC proliferation-related hippo pathway was also activated by lycopene. The underlying mechanism may be that lycopene repressed hypoxia-induced oxidative stress via HIF-1*α*-NOX4-ROS axis in HPH (Figure 9).

Pulmonary vascular remodeling is an important pathological process in HPH due to the hyperproliferation of PASMCs [6, 37]. PASMCs are the main cells in the middle layer of the pulmonary arteriole vasculature, as effector cells for pulmonary vasoconstriction and the cytological basis for pulmonary vascular remodeling [38, 39]. Physiologically, PASMCs are in a dynamic balance between proliferation and apoptosis. Pathologically, PASMCs undergo dedifferentiation and transform into a synthetic phenotype with strong proliferative and migratory abilities [40]. In our study, we found that lycopene inhibited hypoxia-induced PASMC hyperproliferation and reversed the upregulation of Ki67, which was the cell proliferation marker. Likewise, the inhibitory effect of lycopene on PASMC proliferation was demonstrated by EdU staining. We verified that lycopene reduced the percentage of PASMCs in S-phase and remarkably suppressed hypoxia-induced overexpression of cyclin D1 and cyclin D3 and reduction in P27 in PASMCs under hypoxic conditions. Next, we found that hypoxia inhibited PASMC apoptosis by Hoechst staining detection and suppressed the expression of cleaved-caspase 7 and cleaved-caspase 9. Encouragingly, lycopene reversed such a trend. It has been reported that hippo family members regulate the pulmonary hypertension procession and pulmonary vascular remodeling [31–36]. Suppressed Hippo/LATS1 is found in hypoxia-induced rat and mouse PH models and PAH subjects [32]. Inactivated LATS1 enables YAP/TAZ to contribute to the hyperproliferation of PASMCs and pulmonary vascular remodeling [32, 33]. Moreover, MST1 downregulated by several miRNAs leads to PASMCs hyperproliferation under hypoxia conditions [34, 35]. In the present study, lycopene reversed the hypoxia-induced reduction in MST1, LATS1, and MOB1. Moreover, YAP and TAZ, downstream effectors of the hippo signaling pathway, were downregulated by lycopene under hypoxia.

Oxidative stress is a key factor contributing to the structural remodeling of little pulmonary arteries [41–43]. This study observed that lycopene treatment significantly inhibited ROS overproduction induced by hypoxia. ROS was specifically involved in multiple rodents' models of hypoxia-induced pulmonary vascular remodeling and suppression of ROS reversed HPH [27, 44]. In vivo, we demonstrated that hypoxia-induced decrease in SOD and increase in MDA were reversed by lycopene in the lung tissues and serum. SOD protects cells by catalyzing the conversion of superoxide radicals to hydrogen peroxide against potential damage by superoxide radicals [45, 46]. Reduction in SOD expression and/or activity facilitated oxidative stress and vascular remodeling among PH of various etiologies [47]. In addition, increased MDA, a marker of lipid peroxidation, was reported to mediate the pathogenesis and progression of PH. Antilipid peroxidation therapy was effective in the remission of PH [48, 49]. Although several enzymes have been approved to generate ROS, NADPH oxidase is the most significant, closely participating in pulmonary vascular remodeling and vasoconstriction [42]. NOX4 is an important isomer of NOXs [50]. Moreover, NOX4 is confirmed to elevate pulmonary vascular remodeling in the HPH rodent models [51, 52]. It is worth noting that the hyperproliferation and migration of PASMCs are closely related to an increase in NOX4-derived ROS [13, 14, 53]. In agreement with the evidence, the expression of NOX4 at mRNA and protein levels was upregulated in PASMCs under hypoxia conditions, while small interfering RNA- (siRNA-) mediated NOX4 silencing decreased ROS production, cell proliferation, and apoptosis resistance [12, 14, 54]. HIF-1*α* is a highly hypoxia-specific transcription factor. Its activation is a key component of cellular perception and adaptation to the oxygen partial pressure of the internal environment. The HIF-1*α* subunit in vascular smooth muscle plays a significant regulatory role in chronic hypoxia-induced pulmonary vascular remodeling and PH. HIF-1*α* could be bound at the NOX4 promoter hypoxia response element (HRE), thereby enhancing the NOX4 promoter activity in PASMCs under hypoxia conditions [14]. Inferring from this evidence, the NOX4 promoter activity bound downstream of HIF-1*α* may decrease following the decreased expression of HIF-1*α*, resulting in a decrease in NOX4 protein level and ROS production [52, 55]. Although it is well known that ROS activated HIF-1*α* [56], these studies indicated that increased HIF-1*α* appeared to in turn aggravate production of NOX4-derived ROS. In this study, lycopene reversed hypoxia-induced overexpression of HIF-1*α* in protein levels, with a reduction in NOX4 expression and ROS generation. In order to explain whether lycopene can target HIF-1*α* to reduce NOX4 expression and ROS production, we used 2ME2, a HIF-1*α* inhibitor, to treat hypoxic PASMCs. Our study suggested that lycopene inhibited hypoxia-induced oxidative stress via repressing HIF-1*α*-NOX4-ROS axis. And the decreased ROS may further reduce HIF-1*α* activity to alleviate pulmonary vascular remodeling in HPH as previous reported [56]. However, lycopene did not significantly change HIF-1*α* mRNA levels. Further exploration indicated that lycopene promoted proteasome-dependent degradation in HIF-1*α* protein. Based on these data and reports, it is presumed that inhibition of HIF-1*α*-dependent NOX4-derived ROS by lycopene may account for the ability to alleviate oxidative stress in HPH.

## 5. Conclusions

To summarize, lycopene treatment reversed the increased RVSP, RV hypertrophy, and vascular remodeling in the HPH rat model. Lycopene alleviated oxidative stress via repressing HIF-1*α*-NOX4-ROS axis, thereby inhibiting proliferation, restraining migration, and promoting apoptosis of PAMSCs in response to hypoxia. These data offer new clues that lycopene may be a potential compound for PH treatment.

## Data Availability

The data used to support the findings of this study are available from the corresponding author upon request.
