# Correction to “Lycopene Ameliorates Hypoxic Pulmonary Hypertension via Suppression of Oxidative Stress”

**DOI:** 10.1155/omcl/9847903

**Published:** 2026-07-02

D. Wang, Y. Ji, R. Wang, et al., “Lycopene Ameliorates Hypoxic Pulmonary Hypertension via Suppression of Oxidative Stress,” *Oxidative Medicine and Cellular Longevity* 2022, no. 1 (2022): 9179427, https://doi.org/10.1155/2022/9179427.

In the article titled, “Lycopene Ameliorates Hypoxic Pulmonary Hypertension via Suppression of Oxidative Stress,” there were multiple errors. These errors are listed below and corrected as follows:

**Error in the author list:**

The author Miaomiao Gong was omitted from the author list by accident. Their contributions included data collection and analysis. The correct author list should be as follows:

“Dingyou Wang, Yuke Ji^1^, Rui wang^1^, Miaomiao Gong^1^, Ke Cheng^1^, Liang Liu^1^, Na Wu^1^, Qing Tang^1^, Xu Zheng^1^, Junxia Li^1^, Zhilong Zhu^1^, Qinghua Wang^1^, Xueyan Zhang^1^, Runbo Li^1^, Jinjin Pan^1^, Zheng Sui^2^, and Yuhui Yuan^1^.

**Error in the author’s affiliation:**

The details of the affiliation “^1^The Second Affiliated Hospital, Institute of Cancer Stem Cell, Dalian Medical University, Dalian 116000, China” were incorrect.

The correct name of the affiliation should have been written as:

^1^The Second Affiliated Hospital, Institute of Cancer Stem Cell, Dalian Medical University, Dalian 116044, China.

**Errors in the article text:**

The article also contains several textual errors, which occurred during the typesetting process:

*In the Introduction:*

– The World Health Organization (WHO) classified9r into five groups, and hypoxic pulmonary hypertension (HPH) is related to Group 3 [2].
– Should read as:
– The WHO classified PH into five groups, and HPH is related to Group 3 [2].
– Numerous pieces of evidence have demonstrated that oxidative stress contributes to PASMC proliferation in hypoxia‐induced PH when excessive reactive oxygen species (ROS) are generated [10, 11].
– Should read as:
– Numerous pieces of evidence have demonstrated that oxidative stress contributes to PASMCs proliferation in hypoxia‐induced PH when excessive ROS are generated [10, 11].

*In Section 3.3 “Lycopene Inhibited Hyperproliferation and Promoted Apoptosis of Hypoxic PASMCs”:*

– To explore the cellular basis of pulmonary vascular remodeling, the primary PASMC viability was examined by a CCK‐8 assay. The results showed that lycopene reduced hypoxia‐induced PASMC hyperproliferation in a dose‐dependent manner (Figure 3a). Then, lycopene (1 μM) was chosen for further investigation. Hypoxia upregulated the expression of Ki67 in PASMCs and lung tissues; however, the upregulation was lowered after lycopene treatment (Figure 3b–g).
– Should read as:
– To explore the cellular basis of pulmonary vascular remodeling, the primary PASMCs viability was examined by a CCK‐8 assay. The results showed that lycopene reduced hypoxia‐induced PASMCs hyperproliferation in a dose‐dependent manner (Figure 3a). Then, lycopene (1 μM) was chosen for further investigation. Hypoxia upregulated the expression of Ki67 in lung tissues and PASMCs; however, the upregulation was lowered after lycopene treatment (Figure 3b–g).

*In Section “3.6. Lycopene Activated Hippo Signaling Pathway in Response to Hypoxia In Vivo and In Vitro”:*

– However, the changes were reversed following lycopene treatment (Figure 6a,c–g).
– Should read as:
– However, the changes were reversed following lycopene treatment (Figure 6a,c–e,g,h).

*In the Discussion:*

– The PASMC proliferation‐related hippo pathway was also activated by lycopene.
– Should read as:
– The PASMCs proliferation‐related hippo pathway was also activated by lycopene.
– In our study, we found that lycopene inhibited hypoxia‐induced PASMC hyperproliferation and reversed the upregulation of Ki67, which was the cell proliferation marker. Likewise, the inhibitory effect of lycopene on PASMC proliferation was demonstrated by EdU staining. We verified that lycopene reduced the percentage of PASMCs in S‐phase and remarkably suppressed hypoxia‐induced overexpression of cyclin D1 and cyclin D3 and reduction in P27 in PASMCs under hypoxic conditions. Next, we found that hypoxia inhibited PASMC apoptosis by Hoechst staining detection and suppressed the expression of cleaved‐caspase 7 and cleaved‐caspase 9.
– Should read as:
– In our study, we found that lycopene inhibited hypoxia‐induced PASMCs hyperproliferation and reversed the upregulation of Ki67, which was the cell proliferation marker. Likewise, the inhibitory effect of lycopene on PASMCs proliferation was demonstrated by EdU staining. We verified that lycopene reduced the percentage of PASMCs at S‐phase and remarkably suppressed hypoxia‐induced overexpression of cyclin D1 and cyclin D3 and reduction in P27 in PASMCs under hypoxic conditions. Next, we found that hypoxia inhibited PASMCs apoptosis by Hoechst staining detection and suppressed the expression of cleaved‐caspase 7 and cleaved‐caspase 9.
– Suppressed Hippo/LATS1 is found in hypoxia‐induced rat and mouse PH models and PAH subjects [32].
– Should read as:
– Suppressed Hippo/LATS1 is found in hypoxia‐induced rat and mouse PH models and PH subjects [32].

Errors in Figure **8**:

In Figure 8e, the panels “ROS—2ME2 + lycopene” and “ROS—lycopene” were inadvertently duplicated. In addition, the notation indicating a significant difference between “Lycopene” and “Lyc + 2ME2” in Figure 8c is incorrect. The figure legend for Figure 8 is also incorrect.

The correct Figure 8 is as follows:

**Figure 8:**
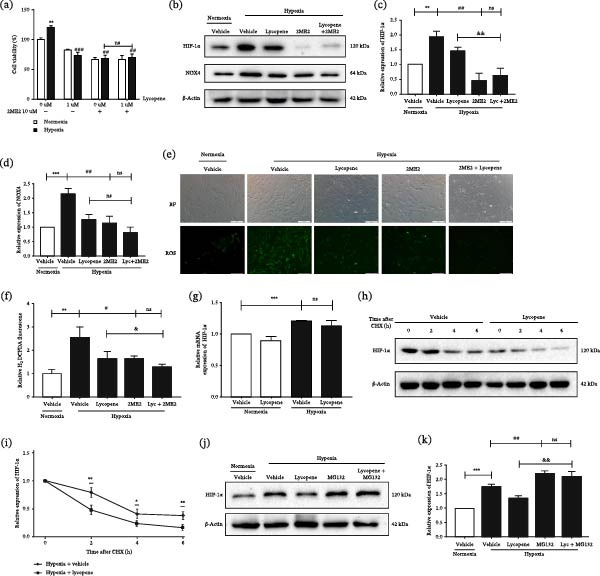
Lycopene regulated HIF‐1α‐NOX4‐ROS axis to inhibit oxidative stress in HPH. (a) The cell viability in PASMCs was detected by CCK8 assay after pretreatment with 2ME2 for 2 h and followed by lycopene for 48 h in response to hypoxia.  ^∗∗^ *p*  < 0.01 vs. the normoxia group; ##*p*  < 0.01, ###*p*  < 0.001 vs. hypoxia group. (b) The expression of HIF‐1α and NOX4 in PASMCs pre‐treated with 2ME2 for 2 h and followed by lycopene for 48 h was detected by western blot. β‐Actin was used as a loading control. (c, d) The quantification of HIF‐1α and NOX4 protein levels in PASMCs.  ^∗∗^ *p*  < 0.01,  ^∗∗∗^ *p*  < 0.001 vs. the normoxia group; ##*p*  < 0.01 vs. the hypoxia group; &&*p*  < 0.01 vs. the hypoxia plus lycopene group. (e, f) The intracellular ROS was detected in PASMCs pretreated with 2ME2 for 2 h and followed by lycopene for 48 h by the DCFH‐DA assay kit.  ^∗∗^ *p*  < 0.01 vs. the normoxia group; #*p*  < 0.05 vs. the hypoxia group; &*p*  < 0.05 vs. the hypoxia plus lycopene group. (g) HIF‐1α mRNA levels were detected by qPCR.  ^∗∗∗^ *p*  < 0.001 vs. the normoxia group. (h) The expression of HIF‐1α in PASMCs was detected by western blot at the indicated time points after adding cyclohexanone (12.5 mg/mL). (i) The quantification of the decay rate of HIF‐1α. 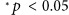,  ^∗∗^ *p*  < 0.01 vs. the hypoxia group. (j) The expression of HIF‐1α in PASMCs were detected by western blot after treatment with MG132 (500 nM) or lycopene for 48 h. (k) The quantification of HIF‐1α protein levels.  ^∗∗∗^ *p*  < 0.001 vs. the normoxia group; ##*p*  < 0.01 vs. the hypoxia group; &&*p*  < 0.01 vs. the hypoxia plus lycopene group. Values are means ± S:E:M (*n* = 3–5). ns, no significance.

We apologize for these errors.

